# CanRestoreFunction: Cancer-related fatigue management eHealth intervention- a pilot pragmatic randomized-control trial

**DOI:** 10.1007/s00520-026-10477-5

**Published:** 2026-02-26

**Authors:** Anne B. Fleischer, Mary Insana Fisher, Wei-Wen Hsu

**Affiliations:** 1 Department of Rehabilitation, Exercise and Nutrition Sciences, University of Cincinnati, 3225 Eden Ave., Cincinnati, Ohio 45267 USA; 2 Department of Physical Therapy, University of Dayton, Dayton, Ohio USA; 3 Department of Biostatistics, Health Informatics and Data Sciences, University of Cincinnati, Cincinnati, Ohio USA

**Keywords:** eHealth, Quality of life, Rehabilitation, Cancer-related fatigue, Occupational therapy, Physical therapy

## Abstract

**Purpose:**

To investigate the effect of an 8-week multi-modal eHealth cancer-related fatigue intervention in women with breast cancer. eHealth included remote education, individualized exercise, and weekly problem-solving sessions. Primary outcomes were recruitment/retention and cancer-related fatigue level. Quality of life was a secondary aim.

**Methods:**

Women with Stage 0–3 breast cancer between 18–70 years who scored ≥ 4/10 on the One-Item Fatigue Scale, completed chemotherapy and/or radiation within 5 years, and did not have chronic fatigue, were randomly assigned to eHealth intervention (eHealth) (*n* = 18) or control (*n* = 24). Cancer-related fatigue and quality of life were measured before and after intervention using the Brief Fatigue Inventory and the Functional Assessment Cancer Therapy-General.

**Results:**

Social media was an effective way to recruit participants into this study; however, more participants in the eHealth group dropped out than the control group. Both groups' fatigue scores significantly decreased over time (*p* = 0.001). eHealth decreases in fatigue reached a minimally clinically important difference (2.25), but control did not (1.3). Quality of life significantly improved in the eHealth group compared to the control (*p* = 0.03).

**Conclusion:**

Multi-modal cancer-related fatigue eHealth intervention appears to clinically reduce fatigue and improve quality of life among women with breast cancer.

US Clinical Trial Register NCT05868187. Date registered: 2–7-2023. www.clinicalgrials.gov

**Supplementary Information:**

The online version contains supplementary material available at 10.1007/s00520-026-10477-5.

## Introduction

In 2025, it is expected over 320,000 women will be diagnosed with breast cancer in the US, which accounts for 32% of all female cancers [1]. Due to the advancements in breast cancer treatment, 91% of these individuals will likely survive 5-years [1]. As the breast cancer death rate has declined by 42% from 1989 to 2021 [1], individuals with breast cancer are expected to regain the same life expectancy as the general population [2, 3]. Unfortunately, these individuals experience considerable side effects. The most common side effect is cancer-related fatigue (CRF), which limits the ability to complete daily activities, resulting in a poorer quality of life. According to the National Comprehensive Cancer Network, CRF is defined as a “distressing, persistent, subjective sense of physical, emotional and/or cognitive tiredness or exhaustion related to cancer or its treatment that is not proportional to recent activity and interferes with usual functioning” [4]. A meta-analysis of 84 research studies of 144,813 cancer survivors estimated the pooled prevalence of CRF to be 52% [5]. However, individual studies have indicated the prevalence to be as high as 75 to 84% [5].

Several studies have investigated non-pharmaceutical CRF interventions. A systematic review by Meneses-Echávez et al. [6] and a metanalysis by Kessels et al. [7] each found that completing an exercise program at least three times a week over an 8-week period consisting of resistance and aerobic training at a level of 40–60% maximum heart rate reduced the physical feelings of CRF. These findings are reflected in the international multi-disciplinary consensus statement for the treatment of CRF [8]. In addition to physical activity, moderate evidence has been found for the use of internet self-management interventions to reduce CRF [9]. These interventions include personalized advice, action planning and problem-solving strategies. Lastly, a systematic review of patient CRF education program randomized control trials (RCTs) [10] found patient education targeting physical exercise, sleep hygiene, relaxation training, and nutrition was associated with significantly less fatigue and anxiety compared to a control group.

One of the modes of healthcare delivery that is becoming more common is eHealth, or electronic health. eHealth refers to information related to healthcare services that are delivered via technological methods such as the Internet and has shown to benefit cancer survivors through improving accessibility of health information and reducing medical costs [11]. eHealth is a term that encompasses multiple different forms of electronic healthcare service delivery, such as telehealth, mHealth (mobile health), and digital applications, and is commonly used to deliver interventions to address cancer symptom management [11].

Several studies have evaluated the impact of psychological and exercise interventions on CRF [12] or single interventions including exercise [13] cognitive behavioral therapy [14] and internet self-management [9]. To our knowledge, no one has evaluated a multimodal eHealth program, including exercise, education and self-management (problem-solving) aimed at addressing CRF. Each intervention has been found to independently be associated with reducing fatigue but has not been investigated collectively. Our pilot randomized trial was designed to evaluate the preliminary efficacy of an eHealth intervention including individualized exercise, problem-solving approach and education. The primary aims of this 8-week pilot eHealth RCT were to determine recruitment and retention rates, evaluate adherence to the intervention, and assess the preliminary efficacy in reducing CRF in women with breast cancer. Our secondary aim was to measure the effect of an eHealth multimodal intervention on quality of life (QOL).

## Methods

### Study design

**Fig. 1:**
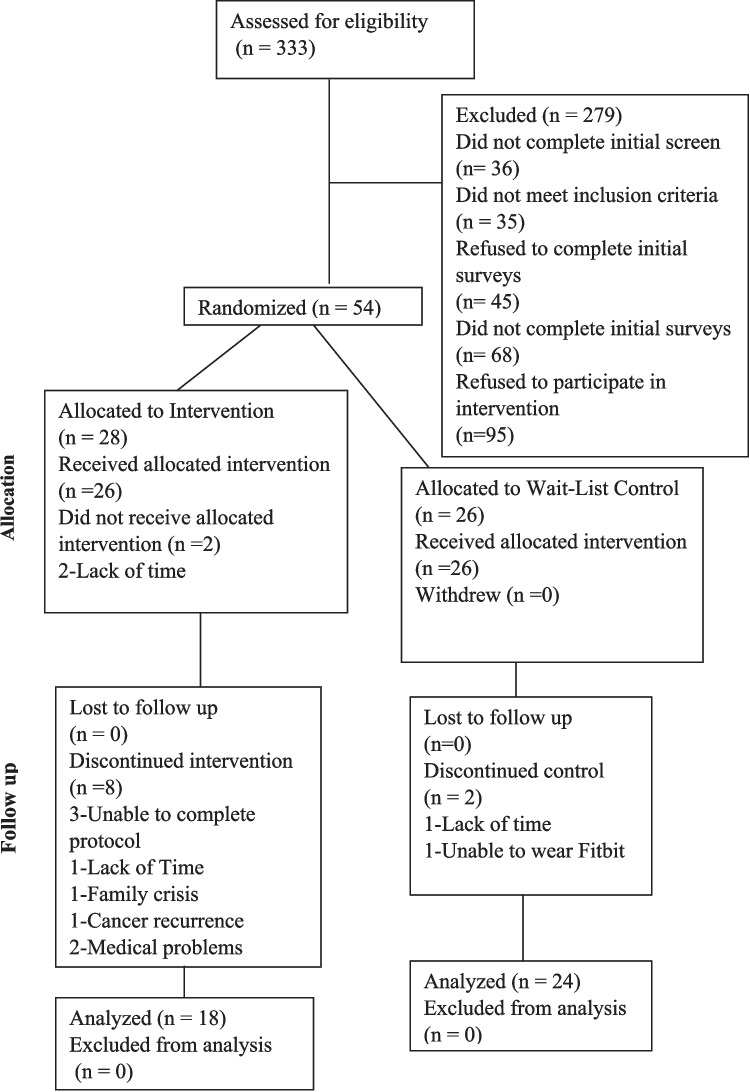
CONSORT diagram

This randomized, waitlist-controlled pilot trial with 1:1 group assignment was conducted in accordance with the Consolidated Standards of Reporting Trials (CONSORT) for randomized pilot and feasibility trials [15] by including items within the CONSORT checklist and illustrating through the CONSORT diagram (Fig. 1). Before the study was initiated, a biostatistician generated a randomization REDCap list. The study groups were assigned sequentially accordingly [16, 17]. Recruitment occurred between March 2022 and July 2024. The study was approved by the University of Cincinnati Institutional Review Board. The study was registered on ClinicalTrials.gov (NCT05868187).

### Sample recruitment, screening and enrollment procedures

Survivors were recruited by posting flyers on university social media (Facebook, Instagram) and within a university cancer center. Scripted emails were sent to cancer care providers, cancer organizations, and cancer support groups encouraging participation. Flyers and emails contained the research team’s contact information and study eligibility. Survivors were included if they were between 18–70 years old, diagnosed with Stage 0–3 breast cancer, completed chemotherapy and/or radiation therapy within the last 5 years, reported moderate to severe fatigue (≥ 4 on One-Item Fatigue Scale), had access to a mobile device or computer, and had Wi-Fi connection. Survivors were excluded if they had metastatic cancer, did not have a signed medical release, reported chronic fatigue prior to cancer diagnosis,and were unable to follow English verbal or written assessment instructions.

After screening for eligibility, survivors completed the following REDCap^1^ online surveys: Demographic and Medical History, Brief Fatigue Inventory, Functional Assessment of Cancer Therapy-General, and the Functional Assessment Chronic Illness-Fatigue. When these were completed, an automated email was sent to the principal investigator to contact the survivor to schedule a videoconferencing meeting to review the informed consent. After consent was obtained and a Medical Release form was received, Fitbit Inspire3 (Fitbit) was mailed to the participant, and a baseline virtual assessment was scheduled. This baseline assessment was completed by a blinded evaluator and included completing the 6-min walk test (6MWT) [18], the 30 s sit-to-stand test (30STS) [19], and the Canadian Occupational Performance Measure (COPM) [20]. In addition, within this visit, each survivor’s Fitbit was connected to a dashboard (Fitabase, San Diego) for monitoring steps and heart rate intensity. After virtual assessment was completed, survivors were randomly assigned to the intervention or the wait-list control group.

### Instruments

All measures including the evaluations guiding the intervention (6MWT, 30STS and COPM) were repeated after 4- and 8-weeks.

#### Outcome measures

##### Brief Fatigue Inventory (BFI)

Fatigue severity and interference were measured with this 9-item scale based on a 0 (no fatigue) to 10 (greatest fatigue) scale, which is a validated fatigue tool with excellent test–retest reliability. Scores of 0–3 are considered no/low fatigue, 4–6 moderate fatigue, ≥ 7 severe fatigue [21].

##### One item fatigue scale

Survivors answered the following question: “How would you rate your fatigue on a scale of 0 to10 with 0 being ‘no fatigue' and 10 being the ‘worst possible fatigue'?” [22].

##### Functional Assessment of Cancer Therapy-General (FACT-G)

This 27-item set of general questions is divided into four primary quality-of-life domains: physical, social/family, emotional and functional well-being. Total scores range from 0–108 with higher scores reflecting a greater quality of life [23]. The FACT-G has acceptable levels of reliability in the breast cancer population [24].

##### Functional Assessment of Chronic Illness Therapy-Fatigue (FACIT-F)

A 13-item FACT-G subscale questionnaire assessing fatigue and its impact on function using a 0 to 5 Likert scale. Total scores range from 0–52 with lower scores representing higher levels of fatigue. Scores of less than 30 indicate clinically significant fatigue. Fatigue subscale has strong internal consistency (α = 0.93–0.95) [25].

### E-Health intervention

#### Exercise

Four different exercise programs (A-D) were pre-recorded by researchers, beginning with a low intensity program with multiple modifications to simple exercises, progressing through a moderate, to higher intensity program with progressively more challenging exercise. Participants were prescribed an exercise program based on their performance on the 6MWT [18] and 30STS [19]. Participant scores were compared to normative data for gender and age for each test, and the lowest performance (6MWT or 30STS) was used for prescription. If an individual scored within normal range, they were assigned Exercise B. However, if they scored ≤ 2SD lower than normal range or ≥ 2SD from the normal range, they were assigned Exercise A or C respectively. Exercise program D was used to progress exercise in those who were making significant gains in the program.

Survivors were instructed to complete 30–45 min individualized exercises three times a week and record (a) their heart rate after the warm-up, aerobics, strengthening and cool down, (b) their rate of perceived exertion using the Borg Rate of Perceived Exertion 6–20 scale and (c) any challenges or successes with their exercises, within an electronic form after each exercise session.

#### Problem-solving session

Each week, survivors completed a virtual problem-solving session. At the beginning of each session, survivors identified one self-care, work/volunteer or leisure activity identified as impaired within the COPM [20]. During the session, the survivor and researcher co-created a weekly goal and strategies to return to participating in the impaired activity. Then they developed a specific action plan using the identified strategies to meet the established weekly goal.

#### Interactive educational modules

Survivors were assigned four interactive educational modules: What is CRF? Rest and Relaxation, Nutrition and Exercise. Researchers monitored the completion of each module and would verbally remind them to complete the next module during the weekly video call.

### Waitlist control group

Survivors allocated to the waitlist control group wore the Fitbit during the day and completed all study measurements at baseline, after 4- and 8-weeks.

### Study procedures

Prior to the first videoconference, survivors were provided with a link to their Google Site, which included (a) tailored exercise video, (b) link to Google Form to record heart rate and perceived rate of exertion while completing assigned exercises, and (c) link to Community Canvas with interactive educational modules. Within the first meeting, survivors were instructed on how to (a) complete their assigned exercises, (b) use their Fitbit to monitor their heart rate (c) record their heart rate and perceived exertion within the Google Form and (d) access their Community Canvas Course. This first meeting was Day 1 of the 8-week study. Survivors were instructed to complete their assigned exercises 3 times each week, complete action plan developed during problem solving session and complete an interactive educational module (every 2 weeks).

#### Monitoring adherence to intervention

##### Exercise

Participants were instructed to complete a Google Form as they were completing their exercises. They recorded heart rate after warm-up, aerobics, strengthening, and cool down. Afterwards, they rated their perceived exertion using the Borg scale and write any comments related to their exercises, such as “my right knee hurt” or “it is too easy.” These were reviewed with the participant during the weekly visit and the number of completed home exercise sessions was recorded. A priori adherence objective was not set given the nature of this feasibility study. Completing at least 2 sessions/week was considered adherent; the number of weeks in which 2 or more sessions were completed was then recorded. We considered 75% (6 weeks) or greater high adherence, 50–69% (4–5 weeks) moderate adherence, and less than 50% (fewer than 4 weeks) poor adherence. At present, there is no established threshold universally held as “good/high” adherence, and despite many citing 80% as that high threshold for positive change, these authors could not presently find evidence to support this [26]. However, studies do show that higher levels of adherence to exercise produce improved results, but these results are not in the same population being studied here [27].

##### Problem-solving session

 Participants virtually met weekly with occupational therapy who performed a structured interview (Online Resource 1). Participants chose one activity identified within the COPM they wanted to complete with less assistance or more easily. Participant discussed why they cannot complete the activity typically. Collaboratively, strategies to overcome these barriers were discussed then a weekly goal developed along with a specific plan for how to achieve it. At the beginning of subsequent sessions, the participant reported what strategies were used and if the weekly goal was met, partially met or not met. Adherence was achieved if the participant attended the weekly session and reported working toward their weekly goal (Online Resource 1).

##### Interactive educational modules

Content for the interactive educational modules developed by an instructional designer was based on the findings from systematic review of cancer-related fatigue education [10]. Specifically, we included National Comprehensive Cancer Network content targeting physical exercise, sleep hygiene, relaxation training and nutrition. Participants were led through the modules by scripts developed by the research team. Participants chose videos, written content, and downloadable documents to learn more about cancer-related fatigue. Adherence was measured by their completion of a pre- and post-survey for each module inquiring what they wanted to learn (pre-survey) and what they planned to apply (post-survey).

#### Sample size calculation

We conducted a power analysis based on data collected from two earlier small feasibility studies [28, 29] and estimated that the effect size ranged from 1.44 to 2.72 with a two-sided paired *t*-test to examine the change in outcome at the baseline and the 8th week. A minimum of 34 (i.e., 17 per group) survivors were needed to achieve a statistical power of 80% at a 0.05 significance level (assuming conservatively that the true effect size is equal to 1). Then it is expected the statistical power is greater than 80% with our final sample size of 42 (eHealth intervention = 18 and control = 24).

### Analysis

Owing to the nature of the longitudinal study design, data were analyzed using linear mixed-effects models coupled with sandwich estimators, which can accommodate the correlations among the repeated measures and alleviate the impacts induced by model misspecifications. To examine the difference between groups over time, the evaluation was conducted on the interaction term of “eHealth Group” and “Time (weeks)” in the mixed-effects models. The two-independent-sample t-test (for continuous variables) and Chi-square test (for categorical variables) were used to evaluate the differences in demographics and breast cancer history between eHealth intervention and control groups. In our study, one participant in the eHealth Intervention group dropped out at the last checking point. Given the small cohort size in our study, no imputation procedures were performed for this timepoint data. Moreover, the linear mixed-effects models are robust to missing data under the less restrictive Missing at Random (MAR) assumption [30], and thus the statistical results remain validated. The significance level was set as 0.05 throughout the whole analysis. All analyses were performed using SAS software (V9.4; SAS Institute Inc., Cary, NC).

## Results

### Sample characteristics

A total of 42 survivors completed the study (*n* = 18 eHealth, *n* = 24 control). See Table 1 for demographics and Table 2 for breast cancer history. No significant differences between the eHealth and control groups were observed.

**Table 1:** Demographics of the study participants

	eHealth Intervention (*N* = 18)	Control (*N* = 24)	*p*-value^±^
Age: Range/X̄(SD)	28–67/47.7 (11.4)	33–64/48.3 (9.2)	0.85
Race/Ethnicity	**(#, %)**	**(#, %)**	
White	14, 77.8%	19, 79.2	0.71
Black	0, 0%	1, 4.2%	
Asian	2, 11.1%	2, 8.3%	
Latin/Hispanic	1, 5.6%	2, 8.3%	
White & Latin/Hispanic	0, 0%	1, 4.2%	
Do not want to answer	1, 5.6%	0, 0%	
Marital			
Single	2, 11.1%	3, 12%	0.55
Married	13, 72.2%	18, 72%	
Long Term	3, 16.7%	2, 8%	
Divorced	0, 0%	2, 8%	
Education			
Highschool	1, 5.6%	0, 0%	0.69
Some College	1, 5.6%	4, 16.7%	
Associate	1, 5.6%	1, 4.2%	
Bachelor	6, 33.3%	9, 37.5%	
Masters	6, 33.3%	8, 33.3%	
Ph.D	3, 16.7%	2, 8.3%	
Employment			
Unemployed	1, 5.6%	2, 8.3%	0.83
< 30 h	3, 16.7%	4, 16.7%	
> 30 h	11, 61.1%	15, 62.5%	
> 40 h	1, 5.6%	0, 0%	
Retired	2, 11.1%	3, 12.5%	

^±^t-test or Chi-square test

**Table 2:** Breast cancer history

	eHealth Intervention (#,%)	Control (#,%)	*p*-value^±^
Cancer Stage			
0	1, 5.6%	1, 4.2%	0.21
1	4, 22.2%	13, 55.2%	
2	11, 61.1%	8, 33.3%	
3	2, 11.1%	2, 8.3%	
Surgery			
Single Lumpectomy	6, 33.3%	13, 54.2%	0.75
Single Mastectomy	3 16.7%	4, 16.6%	
Bilateral Mastectomy	7 31.8%	6, 25%	
Other Breast Surgery	2, 9.1%	1, 4.2%	
Radiation			
< 1 year	3, 25%	7, 35%	0.23
1–3 years	9, 75%	10, 50%	
3–5 years	0,0%	3, 15%	
Chemotherapy			
< 1 year	5, 31.25%	4, 26.7%	0.18
1–3 years	11, 68.75%	10, 66.7%	
3–5 years	0,0%	1, 6.7%	
Yrs since Dx			
< 1 year	2, 11.1%%	4, 16.7%	0.87
1–3 years	14, 77.8%	14, 58.3%	
3–5 years	2, 11.1%	6, 25%	
Total	**18**	**24**	

^±^Chi-square test

### Recruitment and retention

As shown in Fig. 1, of the 268 screened, 11 were not eligible; 2 did not have breast cancer; 1 had been diagnosed > 10 years ago; 1 was older than 70; 2 did not receive chemotherapy or radiotherapy; 2 had metastatic cancer; and 3 had fatigue < 4. Of the 257 who were eligible, 215 did not complete the informed consent. The remaining 52 were randomized to eHealth (*n* = 38) or waitlist control (*n* = 26). We had a higher drop-out rate in the eHealth group (*n* = 10, 36%) compared to the control group (*n* = 2, 4%). Reasons for dropping out of the eHealth group included: inability to complete the protocol (3), missing appointments (1), family crisis (1), cancer recurrence (1), lack of time (1), and medical problems (2).

### Adherence

Survivors’ adherence with interactive education (97%) and weekly problem-solving sessions (99%) were high but was not high for the unsupervised individualized exercise (75%). Adherence was calculated based on comparing what the participant completed versus the minimum target within the protocol.

### Fatigue

The outcomes of interest were summarized by the eHealth and control groups and given in Table 3 and Online Resource 2, 3, & 4. Based on what was initially observed in Table 3, eHealth and control groups’ fatigue decreased over time. We further conducted analyses with linear mixed effects models to examine their statistical significance. The results are given in Table 4. The eHealth and control groups’ fatigue significantly decreased over time for BFI (*p* = 0.001) and FACIT-F (*p* < 0.0001), but fatigue levels were not significantly different between groups over time (BFI *p* = 0.078, FACIT-F *p* = 0.09). However, in a separate analysis, the Minimally Clinical Important Difference for the BFI (a change of 1.33 or > 4/10) [21, 31, 32] was met for the eHealth group (2.48) but not for the control (1.26).

**Table 3:** Summary of outcome measures

		eHealth Intervention *(N* = *18)*	Control *(N* = *24)*	Total (*N* = *42)*
Outcome	Time point	Mean/Median (SD/IQR)	Mean/Median (SD/IQR)	Mean/Median (SD/IQR)
BFI	Baseline	5.77 (1.85)	5.67 (1.54)	5.71 (1.66)
	4 weeks	4.52 (2.24)	4.89 (1.53)	4.73 (1.85)
	8 weeks	3.52 (2.32)	4.21 (2.00)	3.91 (2.14)
FACIT-F	Baseline	24.61 (9.37)	23.88 (6.20)	24.19 (7.62)
	4 weeks	29.17(10.30)	29.42 (7.76)	29.31 (8.82)
	8 weeks	34.89 (8.71)	30.92 (8.78)	32.62 (8.87)
FACT-G	Baseline	67.14 (15.58)	68.54 (13.51)	67.94 (14.27)
	4 weeks	71.56 (16.68)	69.71 (12.38)	70.50 (14.22)
	8 weeks	75.11 (15.30)	70.36 (13.74)	72.44 (14.46)

**Table 4:** The estimated coefficients and results of the linear mixed-effects models for the outcomes of BFI, FACIT-F, and FACT-G

Outcome	BFI Score			FACIT-F			FACT-G		
	estimate	SE^†^	Two-sided *p*-value^‡^	estimate	SE^†^	Two-sided *p*-value^‡^	estimate	SE^†^	Two-sided *p*-value^‡^
Intercept	5.3	1.45	0.0008	22.03	6.00	0.0008	71.82	9.95	< 0.0001
Age (yrs)	−0.0008	0.03	0.98	0.06	0.12	0.62	0.08	0.20	0.7
eHealth Group	0.19	0.56	0.73	−1.29	2.57	0.62	−2.44	4.2	0.56
Time (weeks)	**−0.16**	**0.05**	0.0010*	**0.8**	**0.13**	< 0.001*	0.23	0.18	0.21
eHealth Group × Time (weeks)	−0.11	0.06	0.08	0.39	0.23	0.09	**0.7**	**0.30**	0.03*
Cancer Stage3 (Y/N)	−0.16	0.92	0.86	0.34	5.27	0.95	5.65	6.43	0.38
Radiation treatment (Y/N)	0.43	0.69	0.54	−2.77	3.09	0.37	−7.87	5.35	0.15
Chemo Treatment (Y/N)	0.05	0.55	0.93	2.63	2.54	0.30	−1.67	4.76	0.73

^†^indicates that standard errors are estimated by a sandwich estimator. ^‡^ indicates that the p-values are calculated by using t distribution

*significant at < 0.05

### Quality of life

Quality of life significantly improved in the eHealth group compared to the control group over time (FACT-G *p* = 0.026, see Table 4)*.*

## Discussion

eHealth self-care management intervention is defined as including “at least two of the following components: information provision, goal setting, behavior management and communication” (p. 3436) [33]. To our knowledge, our study was the first self-care management eHealth intervention to include tailored exercise and weekly problem-solving sessions targeting behavior management, communication, and goal setting, and the provision of information through interactive education. Other multimodal interventions targeting CRF have included multiple types of exercise, [6] and psycho- and sleep education, eurythytherapy and anthroposophic painting [34].

This pilot study was successful in recruiting and retaining participants to achieve the intended power. However, we did experience some challenges. The most effective means of recruitment was the use of social media, with survivors reposting the information on their own pages. The incentive of receiving a Fitbit activity tracker was also useful in recruitment. However, more individuals in the eHealth group compared to control dropped out of the study, reflecting the possibility they were only interested in wearing the Fitbit but not completing the intervention. Many survivors completed the initial surveys and signed the informed consent but were unable to complete the medical release form. Survivors have reported “appointment fatigue” [35] and these survivors were fatigued. This extra step may have deterred them from participating. For future studies, we will explore the necessity of using a medical release or developing more streamlined processes. Additionally, we provided a nominal financial incentive for those who completed the visits and assessments, which appeared to support retention. In the future, we may consider providing financial incentives throughout the project rather than only at the end. This may have provided increased incentive for those who “dropped” out of the eHealth group. Despite dropouts, 80% power was achieved.

In evaluating our findings regarding the impact of this multimodal intervention on CRF, we appear to be the first to include a true control group. Other studies evaluating exercise's impact on CRF compared the experimental group to usual care (undefined in most cases) or some other intervention [6, 7, 36]. We are limited in our ability to compare our results with other studies.

We found multimodal eHealth intervention targeting CRF significantly improved quality of life but did not significantly reduce fatigue compared to the control group. This lack of statistical significance for reducing fatigue may be due to the exercise dosing parameters of this study or that problem-solving sessions lacking a specific focus on completing the exercises. In addition, the exercise may not have been performed for as long or at a high enough intensity to effect change in CRF. A recent systematic review and meta-analysis on aerobic exercise impact on CRF found that interventions of at least three times per week for 12 weeks with sessions a minimum of 60 min resulted in decreased CRF among those with CRF [36]. Problem-solving sessions did not consistently focus on performing exercises three times a week for the level of intended intensity, and weekly goals changed weekly to address participant preferences, but participants may have needed more time to reinforce the effective strategies used to achieve weekly goals. This may have led to not integrating the strategies into their daily life; therefore, limiting its impact on reducing fatigue. However, eHealth group’s fatigue reduction did reach a minimally clinically important difference. eHealth group’s mean fatigue decreased 2.25 points resulting in an average fatigue level of 3.52. As the generally accepted definition of CRF is a rating of ≥ 4/10, the intervention group appears to have reduced fatigue below this threshold for CRF definition, while the control group did not reduce its fatigue below 4 [21]. Our fatigue findings are consistent with those found within a systematic review of web-based behavior change interventions targeting cancer related fatigue [11]. Reasons for not reaching significant difference between groups need to be further explored but could include inadequate adherence.

The tailored exercise intervention was based on current evidence of supervised interventions [6, 8]. The survivors’ adherence to exercise was lower (75%) compared to studies with supervised exercise [6]. Although many survivors included exercise within their weekly goals, many still struggled to reach the recommended target for completing exercise 3 times per week. Future eHealth interventions should consider synchronous exercise as an option. Although this is appealing, practically many survivors struggle with having too many appointments. eHealth option provides more flexibility. To support adherence or create a habit to exercise, psychologists describe the importance of associating the desired action (exercise) with a physical location [37] or making it more difficult to complete an activity that prevent exercising (removing social media app) [38] or making it easier to complete exercises (having exercise equipment organized and out) [39]. Behavioral skills such as practicing and receiving feedback on exercise performance and developing a plan to exercise are promising areas to target to improve exercise compliance [40]. Future protocols should intentionally target exercise context and provide behavioral skills training to support exercise and other habits associated with reducing fatigue.

Our study is different from other self-care management RCTs because we included more than one intervention [33]. We found our intervention significantly improved quality of life, perhaps suggesting that individuals with CRF appear to benefit from multimodal interventions rather than a single intervention. Furthermore, our intervention differs from interventions targeting CRF because our survivors developed goals targeting daily activities impacted by fatigue such as work, social participation and leisure rather than goals specific to managing their fatigue. This may be why the participants in our study reported improved quality of life compared to other studies. Future research is needed to understand if utilizing an intervention that targets reducing fatigue or improving ability to complete daily activities impacted by fatigue has a greater impact on perceived fatigue reduction.

### Implications

Our findings suggest that tailored exercise, CRF education, and individualized goals may reduce fatigue and improve quality of life. Although this intervention was delivered remotely, these could be integrated into in person visit. Individualized tailored home exercise does not seem to be as effective as in person. When faced with situation where an individual cannot attend in person, individualized home exercise may be better than no exercise if it is monitored remotely.

### Limitations

There are several limitations to this study. First, 53 (38%) of those eligible consented to participate. We had a higher drop-out rate in the eHealth group (*n* = 10, 36%) compared to the control group (*n* = 2, 4%). We do not know if the participants completed the exercises as instructed since we did not observe them and if participants met their weekly goals because we depended on self-report. Survivors completed the pre- and post-interactive educational module survey; however, we do not know what specific content they reviewed.

## Conclusion

This study supports the feasibility of providing eHealth self-care management including tailored exercise, weekly problem-solving sessions and interactive education to individuals recovering from breast cancer and who have CRF. Within the landscape of changing rehabilitation reimbursement, alternative effective and efficient modes of delivering CRF self-care management interventions are needed, such as eHealth. This study supports alternative intervention methods. Our findings suggest that eHealth intervention improves quality of life and reduces levels of fatigue to below the diagnostic threshold for fatigue, suggesting preliminary effectiveness of this intervention. Further research is needed with larger sample sizes, different types of cancers, and variable demographic populations.

## Supplementary Information

Below is the link to the electronic supplementary material.

Supplementary file1 (PDF 137 KB)

Supplementary file2 (PNG 71.2 KB)

Supplementary file3 (PNG 80.0 KB)

Supplementary file4 (PNG 77.4 KB)

## Data Availability

The data that support the findings of this study are available from the corresponding author upon reasonable request.

## Footnotes

1 Study data were collected and managed using REDCap electronic data capture tools hosted at the University of Cincinnati. REDCap (Research Electronic Data Capture) is a secure, web-based software platform designed to support data capture for research studies, providing 1) an intuitive interface for validated data capture; 2) audit trails for tracking data manipulation and export procedures; 3) automated export procedures for seamless data downloads to common statistical packages; and 4) procedures for data integration and interoperability with external sources.
