# Supplementary material for: CanRestoreFunction: Cancer-related fatigue management eHealth intervention- a pilot pragmatic randomized-control trial
Source: Support Care Cancer. 2026 Feb 26;34(3):252. doi: 10.1007/s00520-026-10477-5 (PMC12945909; doi:10.1007/s00520-026-10477-5)
Supplement: Supplementary file 1 — Supplementary file1 (PDF 137 KB) [file 520_2026_10477_MOESM1_ESM.pdf]

| | |
|---|---|
| Participant Code | |
| 1. Assess how well the problem was solved by last week's action plan: Ask participant: "Tell me about [state last week's action plan] | |
| 2. Identify what made the action plan difficult [described above] (i.e., describe the challenges involved in performing an activity in a specific and objective manner). | |
| 3. Identify what made the action plan easy [described above] (i.e., describe the successes in performing an activity in a specific and objective manner). | |
| 4. State occupational performance area [if participant met the goal or wants to address a different occupational performance deficit, review list from the initial COPM and ask which one the participant would like to work toward] | |
| 5 Identify what makes the activity difficult [listed above] (i.e., describe the challenges involved in performing an activity in a specific and objective manner). | |
| 4/6. Set a goal that is behavioural, observable, achievable, and general. | |
| 5/7 Brainstorm multiple solutions that could help meet the goal. [modify, adapt, restore, do in a different way. . . | |

| 6/8 Identify the advantages and disadvantages (i.e., pros and cons) of each solution and choose a solution. | **Advantages** | **Disadvantages** |
|---|---|---|
| Solution #1 | | |
| Solution #2 | | |
| Solution #3 | | |
| Solution #4 | | |
| **Chosen Solution** | | |
| 7/9. Create and implement an action plan that addresses when and how the solution will be implemented, including what resources might be needed [Plan "A"] | | |
| "Plan B" to address foreseeable barriers to executing the solution. | | |

Online Resource 1: OT Problem-Solving Session Record Form
