# Supplementary figures and images for: CanRestoreFunction: Cancer-related fatigue management eHealth intervention- a pilot pragmatic randomized-control trial
Source: Support Care Cancer. 2026 Feb 26;34(3):252. doi: 10.1007/s00520-026-10477-5 (PMC12945909; doi:10.1007/s00520-026-10477-5)

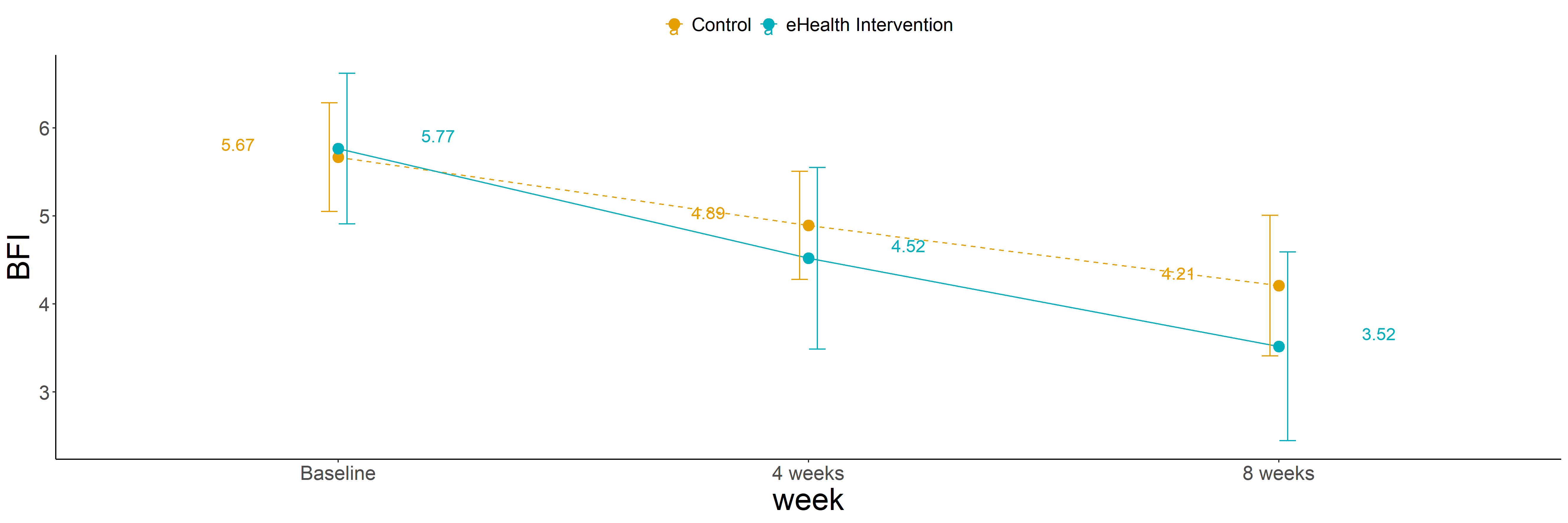

Supplement: Supplementary file 2 — Supplementary file2 (PNG 71.2 KB) [file 520_2026_10477_MOESM2_ESM.png]

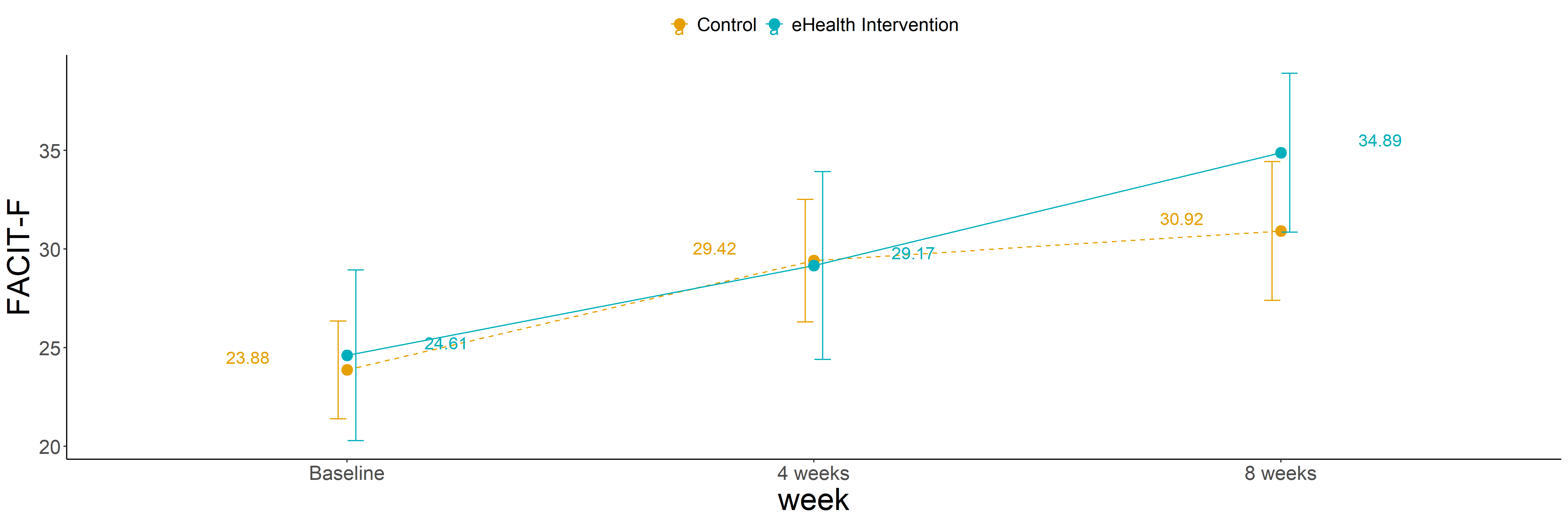

Supplement: Supplementary file 3 — Supplementary file3 (PNG 80.0 KB) [file 520_2026_10477_MOESM3_ESM.png]

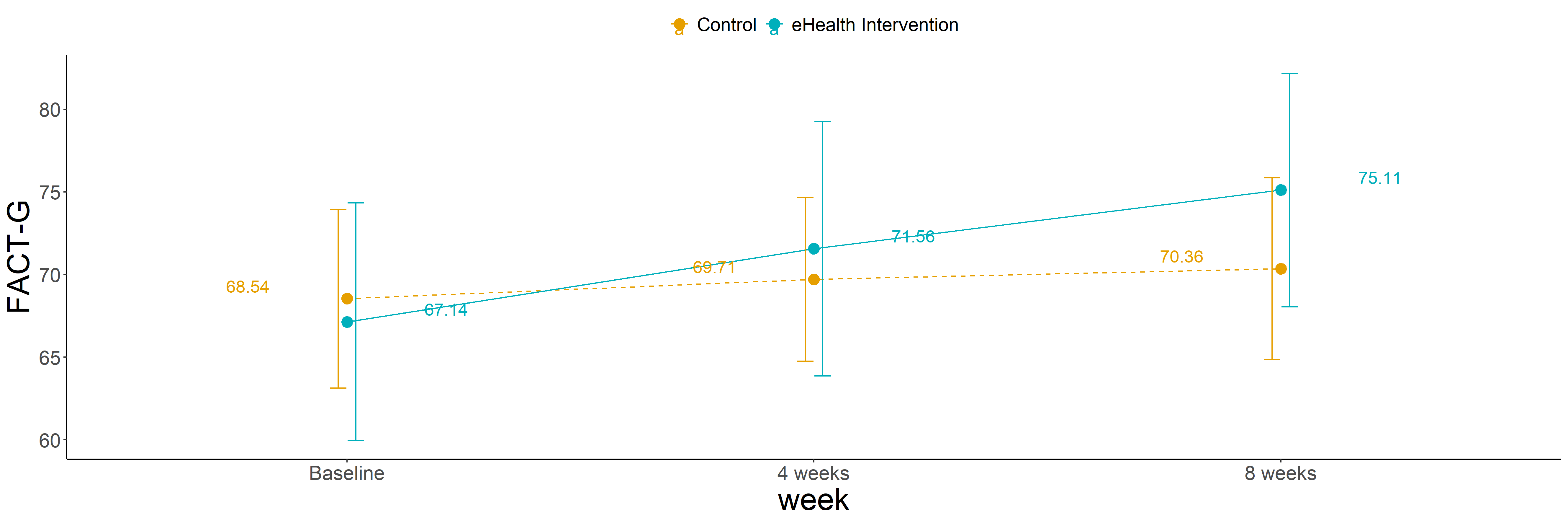

Supplement: Supplementary file 4 — Supplementary file4 (PNG 77.4 KB) [file 520_2026_10477_MOESM4_ESM.png]
